# Psychological distress and coping strategies of hospital nurses during covid-19 pandemic in Greece

**DOI:** 10.1192/j.eurpsy.2024.682

**Published:** 2024-08-27

**Authors:** C. Papathanasiou, K. C. Patithras

**Affiliations:** ^1^Department of Psychology, Panteion University of Social and Political Sciences; ^2^Department of Medicine, National and Kapodistrian University of Athens, Athens, Greece

## Abstract

**Introduction:**

Hospital nurses have played a crucial role during the covid-19 pandemic. Research demonstrates the extent to which nurses were experiencing acute stress and psychological distress during the waves of the pandemic.

**Objectives:**

The purpose of this study was to assess the psychological distress (stress, depression, and anxiety) of nurses working in public hospitals in Greece during the covid-19 pandemic, to identify their coping strategies, and to explore the eventual sociodemographic and work environmental influence on distress and the coping strategies.

**Methods:**

Sample consisted of 317 nurses working in public hospitals. A self-report online questionnaire was used for data collection. The first part of the questionnaire comprised the Depression, Anxiety, and Stress Scale (DASS-21), the second part included the Greek version of the Ways of Coping questionnaire (WAYS), the third part the Oslo Social Support Scale (OSSS-3), and the fourth part included participants’ sociodemographic data. Analyses were conducted using SPSS statistical software (version 26.0).

**Results:**

18.4% of participants presented severe depression, 39.9% very severe anxiety, and 22.5% very severe stress. Significantly lower levels of depression, anxiety, and stress were experienced by those who slept more than 5 hours a day, compared to those who slept up to 5 hours. Participants who were infected with the coronavirus had significantly higher levels of depression, anxiety, and stress. Additionally, participants who received moderate/high social support experienced overall less anxiety, stress, and depression than those who received low social support. Finally, the more they sought social support to deal with their problems and the more they avoided stressful situations, the higher the levels of depression, anxiety, and stress.

**Conclusions:**

A staff care protocol must be applied by every hospital, including rest breaks and night-shift naps, psychosocial support for those who get infected by SARS-CoV-2 and their families, peer support (groups and mentoring), and coping skills trainings.

**Disclosure of Interest:**

None Declared

